# Combined Treatment with Myo-Inositol and Selenium Ensures Euthyroidism in Subclinical Hypothyroidism Patients with Autoimmune Thyroiditis

**DOI:** 10.1155/2013/424163

**Published:** 2013-10-02

**Authors:** Maurizio Nordio, Raffaella Pajalich

**Affiliations:** ^1^University of Rome “Sapienza”, Institute of Gynecology and Obstetrics, Viale del Policlinico, 00155 Rome, Italy; ^2^Ars Medica spa, Via Ferrero di Cambiano Cesare 29, 00191 Rome, Italy

## Abstract

*Background*. Hashimoto's thyroiditis (HT), also known as chronic lymphocytic thyroiditis or chronic autoimmune thyroiditis, is the most common form of thyroiditis affecting more than 10% of females and 2% of males. The present study aims to evaluate the beneficial effect of a combined treatment, Myo-Inositol plus selenomethionine, on subclinical hypothyroidism. *Methods*. The study was designed as a double-blind randomized controlled trial. Eligible patients were women diagnosed with subclinical hypothyroidism having Tg antibodies (TgAb) titer higher than 350 IU/mL. Outcome measures were Thyroid Stimulating Hormone (TSH) levels, thyroid peroxidase antibodies (TPOAb) and TgAb titer, selenium, and Myo-Inositol plasma concentration. *Results*. In the present paper, we demonstrated that the beneficial effects obtained by selenomethionine treatment on patients affected by subclinical hypothyroidism, likely due to the presence of autoantibody (TPOAb and TgAb), are further improved by cotreatment with Myo-Inositol. *Conclusions*. Indeed, due to its action as TSH second messenger, Myo-Inositol treatment reduces TSH levels closer to physiological concentrations.

## 1. Introduction

The pathophysiology of HT can be summarized as follows: triggering of humoral immunity by the abnormal stimulation of T-lymphocytes and consequent destruction of thyroid epithelial cells by chemotaxis, autoantibodies, and inflammatory cascade. Thyrocyte loss is compensated by the increased thyroid-stimulating hormone TSH levels (*Subclinical hypothyroidism*) and the hyperplasia of epithelial cells. Subclinical hypothyroidism is defined as TSH level higher than 4 mIU/L and a normal free-thyroxine level (0.6–1.8 ng/dL) [1]. This condition is associated with an increased risk for coronary heart disease (CHD) events, CHD mortality, and heart failure (HF) events, particularly in patients with TSH levels ≥10.0 mIU/L (for review see [1]). In regions with severe selenium deficiency, there is a higher incidence of thyroiditis due to a decreased activity of selenium-dependent glutathione peroxidase activity within thyroid cells. Selenium-dependent enzymes are also key actors in regulating immune system. Therefore, even mild selenium deficiency may contribute to the development and maintenance of autoimmune thyroid diseases. 

Myo-Inositol is an isomer of a C6 sugar alcohol. Several studies suggested that Myo-Inositol plays an important role in several cellular processes. In particular, it has been demonstrated that Myo-Inositol is the precursor for the synthesis of phosphoinositides, which are part of the phosphatidylinositol (PtdIns) signal transduction pathway [2]. PtdIns is responsible for signal transduction across the plasma membrane, via second messenger: inositol 1,4,5-triphosphate that modulates intracellular Ca^2+^ release or by being a docking site for several signal transduction proteins [3]. 

TSH signaling is rather complex; indeed, two different signal cascades are generated. One branch of the signal cascade involves as second messenger cyclic AMP (cAMP), while another branch is inositol dependent [4, 5]. Indeed, while the cAMP is more involved in cell growth differentiation and the T4-T3 secretion, the inositol-dependent branch regulates H_2_O_2_-mediated iodination [4]. In particular, it has been shown that relatively low TSH concentrations are able to stimulate cAMP mediated signal cascade, while only 100-fold higher TSH concentrations are able to stimulate the inositol-mediated signal cascade [6].

The aim of this study was to evaluate whether the association between Myo-Inositol and Selenium can ensure euthyroidism in subclinical hypothyroidism patients with autoimmune thyroiditis.

## 2. Materials and Methods

The present study was performed as a prospective randomized double-blinded, controlled study in women (*n* = 48; mean age 38 years) with autoimmune thyroiditis and TPOAb; inclusion criteria were TgAb and /or TPOAb above 350 IU/mL, TSH levels between 4,01 mIU/L and 9,99 mIU/L, and a normal free-thyroxine level (0.6–1.8 ng/dL) as well as typical hypoechogenicity of the thyroid in high-resolution sonography. The primary endpoint of the study was restoration of TSH levels (lower than 4 mIU/L). Secondary end points were decreased in serum TPOAb and TgAb concentrations, free thyroid hormone levels and improvement of the thyroid and quality of life estimation. All patients enrolled signed an informed consent. Patients were randomized into 2 groups according to their initial TPOAb concentrations. group A consisted of 24 patients who received orally 83 *μ*g selenomethionine/day, in a soft gel capsule; group B consisted of 24 patients who received a combined treatment plus Myo-Inositol 600 mg also in a 83 *μ*g selenomethionine, soft gel capsule, orally, for 6 months. The patients were asked to take the medication with water about 2 h before or after a meal. They were not given further treatment, such as over-the-counter vitamins or trace elements. All patients were otherwise healthy. No patients were substituted with L-T4. TPOAb, TgAb, TSH, and free thyroid hormones were determined by commercial assays. The echogenicity of the thyroid was monitored with high-resolution ultrasound. 

### 2.1. Laboratory and Technical Investigations

A total of 48 patients were enrolled in the study: two were excluded because they become pregnant during the study period. Twenty-four patients received combined treatment with Myo-Inositol 600 mg plus 83 *μ*g selenomethionine/die, soft gel capsule; twenty-two received soft gel capsule of 83 *μ*g selenomethionine/die. The mean age in both groups was identical.

Blood samples were drawn at the beginning and at the end of the treatment. Free T4 and T3 concentrations and TSH were measured by an enzyme immunometric assay (Byk-Sangtec Dietzenbach, Germany). Plasma total TPOAb and TgAb concentrations were measured by a commercial chemiluminescence assay (Byk-Sangtec). The specificity for autoimmune thyroiditis in these assays is greater than 90% when antibody concentrations are above 350 IU/mL. Plasma selenium was determined by atomic absorption spectrometry. Plasma Myo-Inositol was determined using Gas Chromatography-Mass Spectrometry (GC-MS) analysis after extraction with organic solvents and derivatization. Injection (1.0 *μ*L) was performed on splitless mode at 270°C and a capillary column Agilent 122–5532 DB-5ms (0.25 mm × 30 m × 0.25 *μ*m) was used. Total run time was 15 minutes: oven at 70°C from 0 to 1 min; 20°C/min to 150°C; 10°C/min to 240°C; 4 min at 320°C in postrun. The flow rate was fixed to 1.2 mL/min and results were analyzed by an MS 5973 Network Series detector in sim mode. High-resolution ultrasound (7.5 MHz; SONOLINE Elegra, Siemens, Erlangen, Germany) of the thyroid gland was performed, and echogenicity as well as perfusion by Doppler sonography was documented and compared at the beginning and end of the study by an independent experienced investigator. The subjective wellbeing was evaluated using the standardized SF 12 protocol before and after the study. The SF 12 protocol is a 12-item short form to survey health status in medical outcome studies.

### 2.2. Statistics

The relative changes in antibody concentrations as well as thyroid hormone concentrations in both groups were compared using Wilcoxon's matched pairs, signed-ranks test. In addition, the differences in antibody concentrations at the beginning and end of the study were determined by *t*-test for paired samples. The *P* values were corrected for the numbers of tests performed. 

## 3. Results

At study entry mean TSH levels concentrations free T4, free T3, TgAb, and TPOAb concentrations were not statistically different in both groups (Table 1). The ultrasound pattern in all patients revealed the typical hypoechoic thyroid tissue. None of the patients had thyroid nodules.

Improvement of ultrasound echogenicity was observed in all patients in group B versus ten patients in group A (*P* < 0.01). Evaluation of subjective wellbeing revealed an improvement in 18 patients in group B compared with 8 in group A, no change in 6 patients in group B versus 14 in group A (*P* < 0.05).

Plasma selenium values were identical in both groups at study entry (127.4 ± 15.3 *μ*g/L group A; 129.2 ± 15.1 *μ*g/L group B). In both groups plasma selenium concentration increased, after treatment, by than 75%, precisely in the selenomethionine-treated group selenium levels reached 223.5 ± 15.3 *μ*g/L (*P* < 0.01 versus baseline), while in the combined therapy selenium levels reached 225.4 ± 12.5 *μ*g/L (*P* < 0.01 versus baseline).

As expected, plasma Myo-Inositol level significantly increased, only in the combined therapy group (22.2 ± 4.1 *μ*mol/L versus 37.3 ± 4.5 *μ*mol/L) while there were no differences between the two groups at baseline.

TSH concentrations significantly decreased in group B by 31% (4.4 ± 0.9 versus 3.1 ± 0.6 mIU/mL, *P* < 0.01). On the other hand, there was no change in the TSH level in the group A.

Autoantibody titer, TPOAb and TgAb, significantly decreased in both groups. In particular, TPOAb concentration decreased significantly in the group A by 42% (905.6 ± 401.6 versus 522.6 ± 236.8 mIU/mL, *P* < 0.01) and TgAb decreased by 38% (1080.8 ± 485.1 versus 670.1 ± 300.8 mIU/mL, *P* < 0.01). In group B, TPOAb decreased by 44% (913.9 ± 543.9 versus 516.1 ± 315.4 mIU/mL, *P* < 0.01) and TgAb decreased by 48% (1019 ± 374.2 versus 533.9 ± 258.4 mIU/mL, *P* < 0.01).

Eleven patients in the combined treated group showed a reduction of the TgAb below the threshold identified as inclusion criterion, compared to three patients in group A. Ultrasound of the thyroid showed normalized echogenicity in these patients.

## 4. Discussion

In the present study, we were able to demonstrate that, in subclinical hypothyroidism, patients with autoimmune thyroiditis, treated with Myo-Inositol and selenomethionine, experience a reduction of the increased TSH that selenomethionine supplementation alone was not able to promote. Concomitantly, the concentration of the two autoantibodies declined in both groups. 

Myo-Inositol is a precursor for many inositol-containing compounds that play critical and several roles in signal transduction, membrane biogenesis, vesicle trafficking, and chromatin remodeling [7]. Indeed, many studies support the notion that MI is one of the precursors for the synthesis of phosphatidylinositol polyphosphates (PIPs) that are a source of a number of second messengers. These messengers include diacylglycerol, which regulates some members of the protein kinase C family, inositol-1,4,5-triphosphate, which modifies intracellular calcium levels, and phosphatidylinositol-3,4,5-phosphate, which is involved in the signal transduction [7–9]. Myo-Inositol is a component of cell membranes and plays an important role in cell morphogenesis and cytogenesis, lipid synthesis, structure of cell membranes, and cell growth. Related to all of these signaling pathways, Myo-Inositol is initially incorporated at the level of cell membranes as phosphatidyl-myo-inositol, the precursor of inositol triphosphate, which functions as second messenger regulating the activities of several hormones such as TSH, Follicle-Stimulating Hormone, and insulin [10, 11].

Selenium-dependent enzymes have diverse effects not only within the thyroid [12, 13], but also on the immune system [14–17]. It has been shown that, during severe selenium deficiency, the lack of GPx (selenium-dependent enzyme) activity may contribute to oxidative damage of the thyroid cell and initiation of thyroid damage and fibrosis [18]. Selenium supplementation in a rat model could prevent this oxidative damage [19]. It can be speculated that, even in mild selenium deficiency, this mechanism is an important environmental factor initiating or maintaining autoimmune thyroiditis in people genetically prone to the development of organ specific autoimmunity. The immune modulatory effects of selenium-dependent enzymes such as GPx and TxR are involved in the organ-specific immune response [20]. This was demonstrated in selenium-deficient mice, where lung tissue damage was significantly increased after virus infection compared with selenium-adequate mice [21]. Selenium-dependent enzymes are both antioxidative and anti-inflammatory [14, 22, 23]. This is because GPx can reduce hydrogen peroxides and lipid and phospholipid hydroperoxides, thereby lowering the propagation of free radicals and reactive oxygen species. Lower hydroperoxide tissue concentrations diminish the production of inflammatory prostaglandins and leukotriene. The respiratory burst is also dampened by selenium-dependent enzymes as well as superoxide production [20]. Although tissue damage after viral infection is not comparable to organ-specific autoimmunity, this study clearly demonstrates the striking effects of different nutritional selenium supply on the immune response [17]. 

This mechanism may also contribute to reduced inflammatory activity in the organ-specific autoimmune response [15, 24] and may explain the improvement of autoimmune thyroiditis in our study. In a nonblinded pilot study, significant decreases in TPOAb and thyroid binding inhibitory immunoglobulins, but not TgAb, concentrations were described in patients with Hashimoto's thyroiditis and Graves' disease [24], in accordance with our findings in patients with autoimmune thyroiditis. The clinical benefit of selenium supplementation was also shown in double-blinded studies in patients with rheumatoid arthritis [25] or asthma [26]. In Crohn's disease, plasma selenium and GPx activities are inversely correlated with the activity of the disease [27]. We did not find any alterations in thyroid function after selenium supplementation. This might be due to the fact that the selenium deficiency was only moderate, and deiodinase activity decreases only in severe selenium deficiency [14]. In a previous study in a small cohort of patients with reduced thyroid iodine organification after subacute thyroiditis or postpartum thyroiditis [28], supplementation selenium had no effect on thyroid hormone synthesis. The thyroid is one of the organs with the highest selenium concentration [29], but during mild selenium deficiency deiodinase activities are unaltered, in contrast to GPx activities. Therefore, in tissue samples from patients with autoimmune thyroiditis and nontoxic goiter, there was no difference in selenium tissue concentration in selenium sufficient areas [30]. The selenium deficiency in our patients was mild (0.89 mol/liter), but it is known that in individuals with such low plasma selenium concentrations GPx activity is impaired. The mean plasma selenium concentration necessary for optimal GPx activities is 1.20 mol/liter (range, 1.12–1.44 mol/liter) [31]. This might explain the anti-inflammatory activity of selenium without affecting thyroid hormone levels. We also determined quality of life in our study population. The change in antibody concentrations or inflammatory activity within the thyroid of course has no impact on quality of life, but there are studies showing that low selenium intake is associated with a significant greater incidence of negative mood states and depression [32, 33]. Patients receiving selenium supplementation reported significantly better wellbeing in our trial compared with the placebo group, which supports these earlier findings. The cause is unknown, but there are indications that selenium is important for brain function. The turnover rate of some neurotransmitters is altered in selenium deficiency [34], and low plasma selenium concentrations are associated with senility and cognitive decline [35]. 

The beneficial effect obtained by Myo-Inositol is easily explained by its biological role in signaling TSH hormone; indeed, inositol regulate H_2_O_2_-mediated iodination [4] and it has been shown that hypothyroidism can be caused by an impairment of the inositol-depended TSH signaling branch (TSH resistance) [5]; therefore, by increasing the amount of the second messenger, we can increase the TSH sensitivity. 

In conclusion, in the present paper we demonstrated that the beneficial effects obtained by selenomethionine treatment on patients affected by subclinical hypothyroidism, likely due to the presence of autoantibody (TPOAb and TgAb), are further improved by cotreatment with Myo-Inositol. Indeed, due to its action as TSH second messenger, Myo-Inositol treatment reduces TSH levels closer to physiological concentrations.

## Figures and Tables

**Table 1:** Values are listed as mean ± SD.

	MI-SEL (GROUP B)	SEL (GROUP A)	*P* value
Age	37.95 ± 2.16	38.03 ± 1.63	NS
TSH	4.43 ± 0.89	4.33 ± 0.91	NS
Free T4	18.87 ± 4.02	18.75 ± 3.89	NS
Free T3	6.49 ± 2.36	6.60 ± 1.66	NS
TgAb	1019.74 ± 374.21	1080.80 ± 485.1	NS
TPOAb	913.9 ± 543.9	905.6 ± 401.6	NS
